# Modified Suture‐Gripping Technique for Arthroscopic Meniscal Suturing

**DOI:** 10.1002/atn2.70374

**Published:** 2026-10-28

**Authors:** Ryo Sasaki, Naoki Muroya, Taichi Nishimura, Teppei Hayashi, Kazuya Kaneda, Masaki Nagashima, Shu Kobayashi, Hideo Morioka

**Affiliations:** ^1^ Department of Orthopaedic Surgery NHO Tokyo Medical Center Meguro‐ku Tokyo Japan; ^2^ Department of Orthopaedic Surgery Keio University School of Medicine Shinjuku‐ku Tokyo Japan; ^3^ Department of Orthopaedic Surgery International University of Health and Welfare Mita Hospital Minato‐ku Tokyo Japan

## Abstract

Meniscal preservation is essential for the maintenance of knee function. The suture‐gripping technique, using a suture shuttle, enables accurate needle insertion from inside the joint and flexible withdrawal of the suture from outside the capsule, thereby facilitating meniscal repair of the anterior segment. Using a continuous suturing technique with this method is possible, and we described a modified approach. We recently recognized that ARTHRO‐PIERCE, a device originally designed for hip arthroscopy, can be used to perform the same suture‐gripping technique principles for meniscal repair. The ARTHRO‐PIERCE has a sharper tip—when compared with a suture shuttle holding a suture at its tip—that improves penetration, a more rigid shaft that improves reach and trajectory toward the anterior horn and anterior body, and robust suture grasping without relying on a loop mechanism than a conventional suture shuttle. Here, we describe the step‐by‐step surgical technique. This technique expands the versatility of the suture‐gripping technique for anterior meniscal suturing with improved device handling and secure suture control.

## Graphical abstract

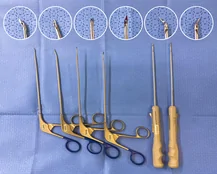

**VIDEO 1:** The suture‐gripping technique (SGT) was introduced to allow accurate inside‐out needle placement from inside the joint while enabling flexible suture withdrawal from outside the capsule. In a previous study, the SGT was performed using a suture shuttle (ACCU‐PASS Suture shuttle; Smith & Nephew Endoscopy, Andover, MA) and was used in both interrupted and continuous meniscal suturing. In the present technical note, we describe a modification of the SGT using ARTHRO‐PIERCE (Smith & Nephew Endoscopy). This device provides a functionally sharper piercing profile—when compared with a suture shuttle holding a suture at its tip—and increased rigidity, which may improve access to the anterior segment and facilitate secure suture grasping. ARTHRO‐PIERCE was introduced as an inside‐out passer. The distal tip geometry varies by model (direction of curvature and bend angle), allowing the selection of the most suitable device according to the intended meniscal repair site. Closing the jaws of the ARTHRO‐PIERCE securely grasps the suture, and opening the jaws releases the suture. The patient was a 44‐year‐old woman who injured her right knee 1 month before presentation. Magnetic resonance imaging revealed a bucket‐handle tear in the lateral meniscus (LM) of the right knee. Under general anesthesia, the patient was placed in a supine position on an operating table with a standard leg holder, allowing a full range of motion. First, standard arthroscopy was performed through the anterolateral portal. The LM had a bucket‐handle tear; hence, the displaced LM was reduced, and the posterior body of the meniscus and posterior joint capsule were sutured using the all‐inside technique, with the knee in the figure‐4 position. In the anteromedial portal view, with the knee in the figure‐4 position, a longitudinal tear was observed on the unstable LM. The tear was sutured through ARTHRO‐PIERCE‐assisted SGT. First, in the anteromedial portal view, with the knee in the figure‐4 position, the ARTHRO‐PIERCE is inserted without gripping a suture into the meniscotibial capsule and the extra‐articular space from the far‐medial portal. When the instrument is advanced through the skin, a small skin incision is made at the planned exit site of the ARTHRO‐PIERCE. Next, a suture (ULTRABRAID Suture; Smith & Nephew Endoscopy) is grasped by the ARTHRO‐PIERCE in the extra‐articular space, and the ARTHRO‐PIERCE is withdrawn, gripping the suture, into the joint space. Next, the ARTHRO‐PIERCE is inserted, gripping the suture into the meniscus and extra‐articular space. The suture is released, and the ARTHRO‐PIERCE is withdrawn into the joint space. Three threads were created for LM suturing. After completing the desired suture, with the knee in the figure‐4 knee position, the anterior portion of the LM is visualized from the anterolateral portal, appropriate tension is achieved, and the suture is tied. Finally, the stability of the LM is assessed and confirmed. Video content can be viewed at https://doi.org/10.1002/atn2.70374.

The meniscus contributes to joint stability, shock absorption, load transmission, proprioception, and cartilage nutrition; therefore, repair and preservation are preferred whenever feasible.^1^ Various arthroscopic repair techniques have been described, including all‐inside, outside‐in, and inside‐out approaches.^1^ Suturing the anterior horn and anterior body can be challenging because outside‐in methods often require complex intra‐articular suture management and are limited by the diameter of the needle, whereas all‐inside devices leave knots or implants within the joint, with potential cartilage irritation.^2^, ^3^

The suture‐gripping technique (SGT) was introduced to allow accurate inside‐out needle placement from inside the joint while enabling flexible suture withdrawal from outside the capsule.^4^ In a previous study, the SGT was performed using a suture shuttle (ACCU‐PASS Suture shuttle; Smith & Nephew Endoscopy, Andover, MA) and was used in both interrupted and continuous meniscal suturing—the hybrid reversible SGT.^5^

However, when a suture shuttle holds a suture at its tip, the tip becomes relatively blunt, which can make tissue penetration technically demanding. In addition, the relative flexibility and limited rigidity of the suture shuttle may compromise precise trajectory control, especially when attempting to reach the anterior horn.

In the present technical note, we describe a modification of the SGT using ARTHRO‐PIERCE (Smith & Nephew Endoscopy). Compared with a suture shuttle holding a suture at its tip, this device provides a functionally sharper piercing profile and increased rigidity, which may improve access to the anterior segment and facilitate secure tissue penetration. We herein present the indications, step‐by‐step techniques, pearls and pitfalls, and advantages and limitations of the technique (Table 1 and Video 1).

**TABLE 1:** Pearls and Pitfalls

Pearls	Pitfalls
Use a far‐medial portal to optimize the trajectory toward the anterior meniscal portion	Incorrect portal placement may cause poor needle trajectory and iatrogenic cartilage injury
Make a small skin incision at the emergence site and dissect to the capsule to safely identify the instrument tip	Inadequate exposure may lead to blind grasping and risk to surrounding soft tissues
Maintain adequate distance between entry points to prevent meniscal cutout	Entry points too close together may result in tissue cut‐through
Confirm secure suture grasp before withdrawing the instrument into the joint	Incomplete suture grasp may cause suture loss during withdrawal
Adjust tension arthroscopically before final knot tying	Over‐ or undertensioning may compromise healing or cause extrusion

## SURGICAL TECHNIQUE

### ARTHRO‐PIERCE‐Assisted SGT

ARTHRO‐PIERCE (Smith & Nephew Endoscopy) is introduced as the inside‐out passer (Figures 1 and 2). After the instrument tip is advanced from the intra‐articular space through the meniscus/capsule into the extra‐articular space, the suture is grasped securely at the instrument tip outside the joint. The instrument is then withdrawn back into the joint while maintaining the grasp. The instrument is reinserted through the meniscus at the second point, advanced outside again, and the suture is released to complete the passage of one suture limb. This process can be repeated to place additional sutures as required.

**FIGURE 1:**
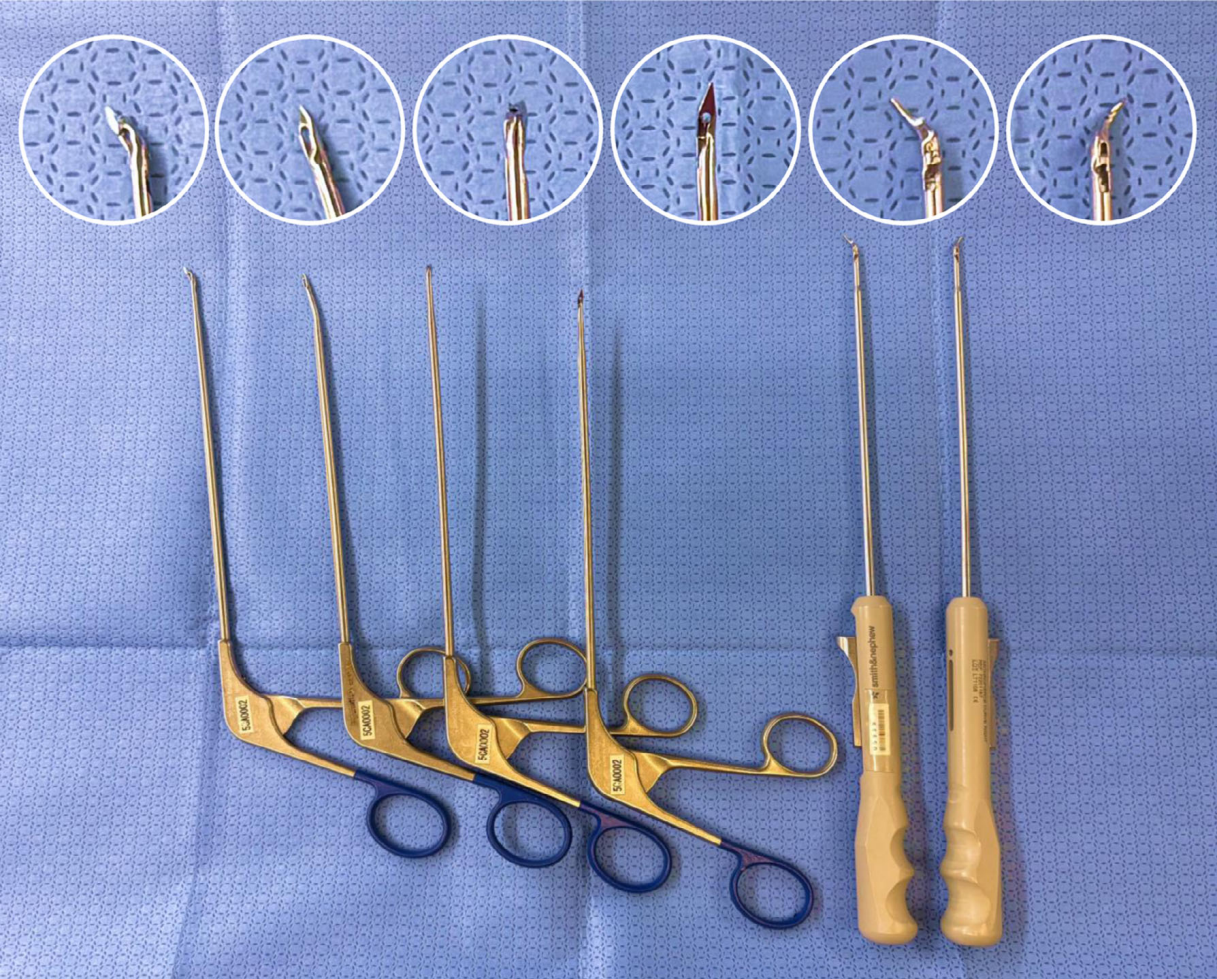
Available ARTHRO‐PIERCE configurations. The distal tip geometry varies by model (direction of curvature and bend angle), allowing the selection of the most suitable device according to the intended meniscal repair site.

**FIGURE 2:**
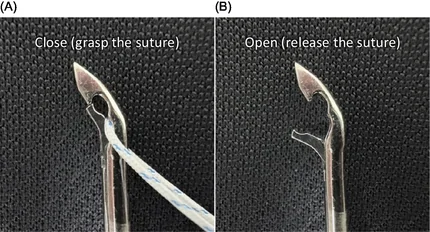
Distal tip configuration of the ARTHRO‐PIERCE device. (A) Closing the jaws securely grasps the suture. (B) Opening the jaws releases the suture.

### Patient and Preparation

Preoperative magnetic resonance imaging is used to evaluate the tear pattern and repairability (Figure 3). Under general anesthesia, the patient is placed in the supine position with a standard leg holder, allowing a full range of motion. Standard arthroscopy is performed through anterolateral and anteromedial portals. For lateral meniscus (LM) anterior suturing, the knee is typically positioned in the figure‐4 position to improve access and visualization.

**FIGURE 3:**
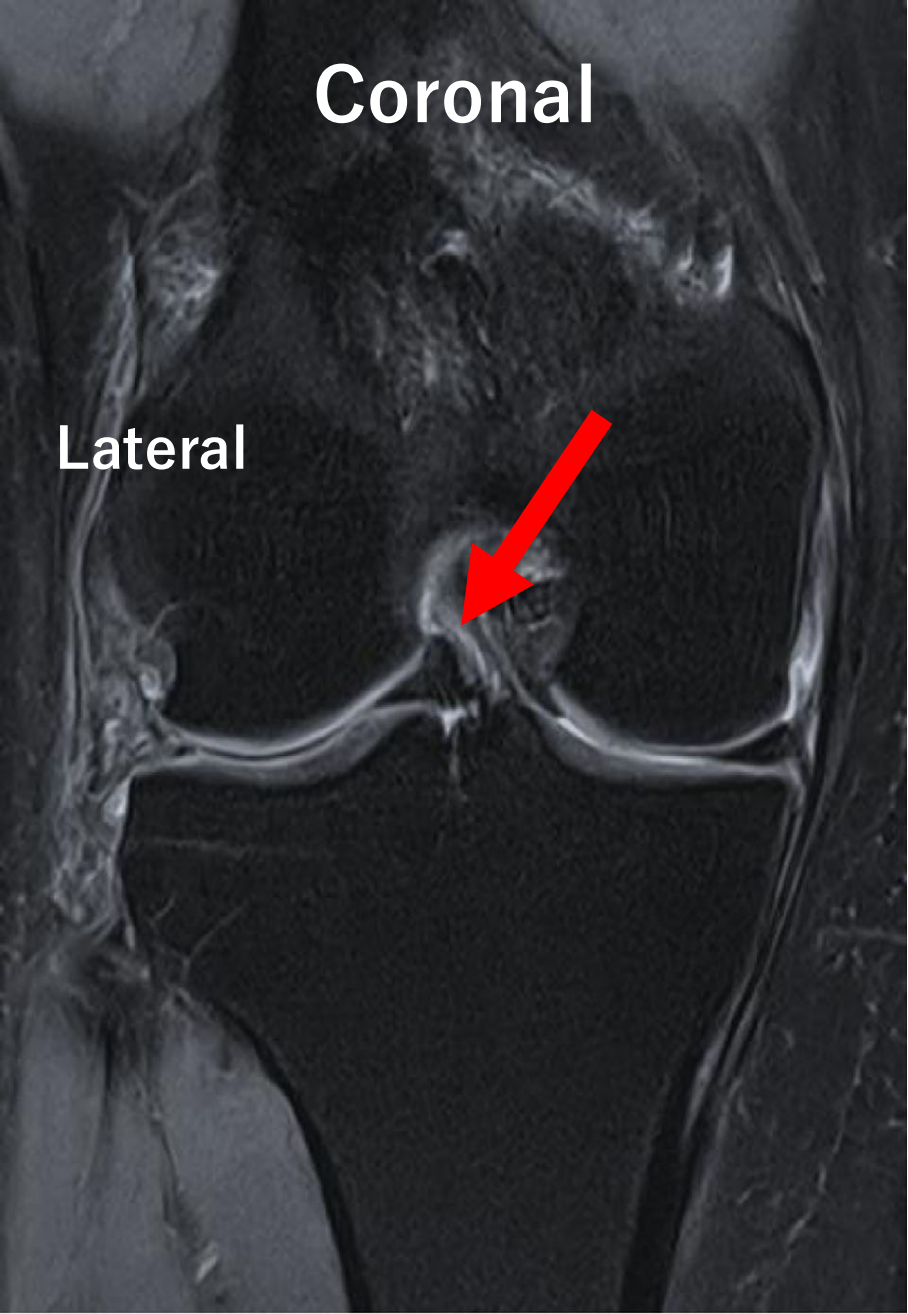
Preoperative magnetic resonance imaging. Coronal imaging shows a bucket‐handle tear of the LM (arrow) in the right knee. (LM, lateral meniscus.)

The LM has a bucket‐handle tear; therefore, the displaced LM is reduced, and the posterior body of the meniscus and the posterior joint capsule are sutured using the all‐inside technique (FAST‐FIX FLEX; Smith & Nephew Endoscopy) with the knee in the figure‐4 position. Next, the longitudinal tear from the anterior to the middle body of the LM is sutured using ARTHRO‐PIERCE‐assisted SGT.

### First Insertion of the ARTHRO‐PIERCE (Without Gripping a Suture)

First, a far‐medial portal is created to obtain an appropriate trajectory for the anterior portion of the LM and visualize the anterior portion of the LM from the anterolateral portal in the figure‐4 position. The ARTHRO‐PIERCE is inserted without gripping a suture into the meniscotibial capsule and extra‐articular space from the far‐medial portal (Figure 4A‐C). When the instrument is advanced through the skin, a small skin incision is made at the planned exit site of the ARTHRO‐PIERCE.

**FIGURE 4:**
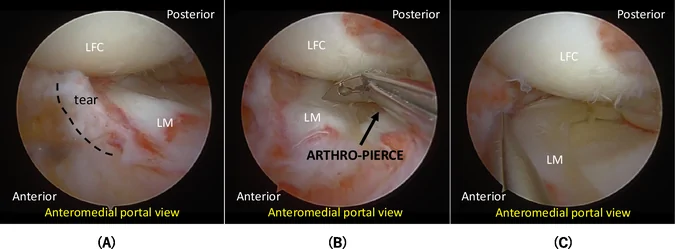
First insertion of the ARTHRO‐PIERCE (right knee, supine and figure‐4 position, arthroscopic anteromedial portal views). The longitudinal tear of the anterior body of the meniscus is diagnosed (A), and the ARTHRO‐PIERCE is inserted into the meniscotibial capsule and extra‐articular space from the far‐medial portal (B and C). (LFC, lateral femoral condyle; LM, lateral meniscus.)

### Gripping the Suture Outside the Joint and Withdrawing Into the Joint Space

The suture (ULTRABRAID Suture; Smith & Nephew Endoscopy) is gripped in the extra‐articular space using ARTHRO‐PIERCE; then, the ARTHRO‐PIERCE is withdrawn while gripping the suture into the joint space (Figures 5 and 6A).

**FIGURE 5:**
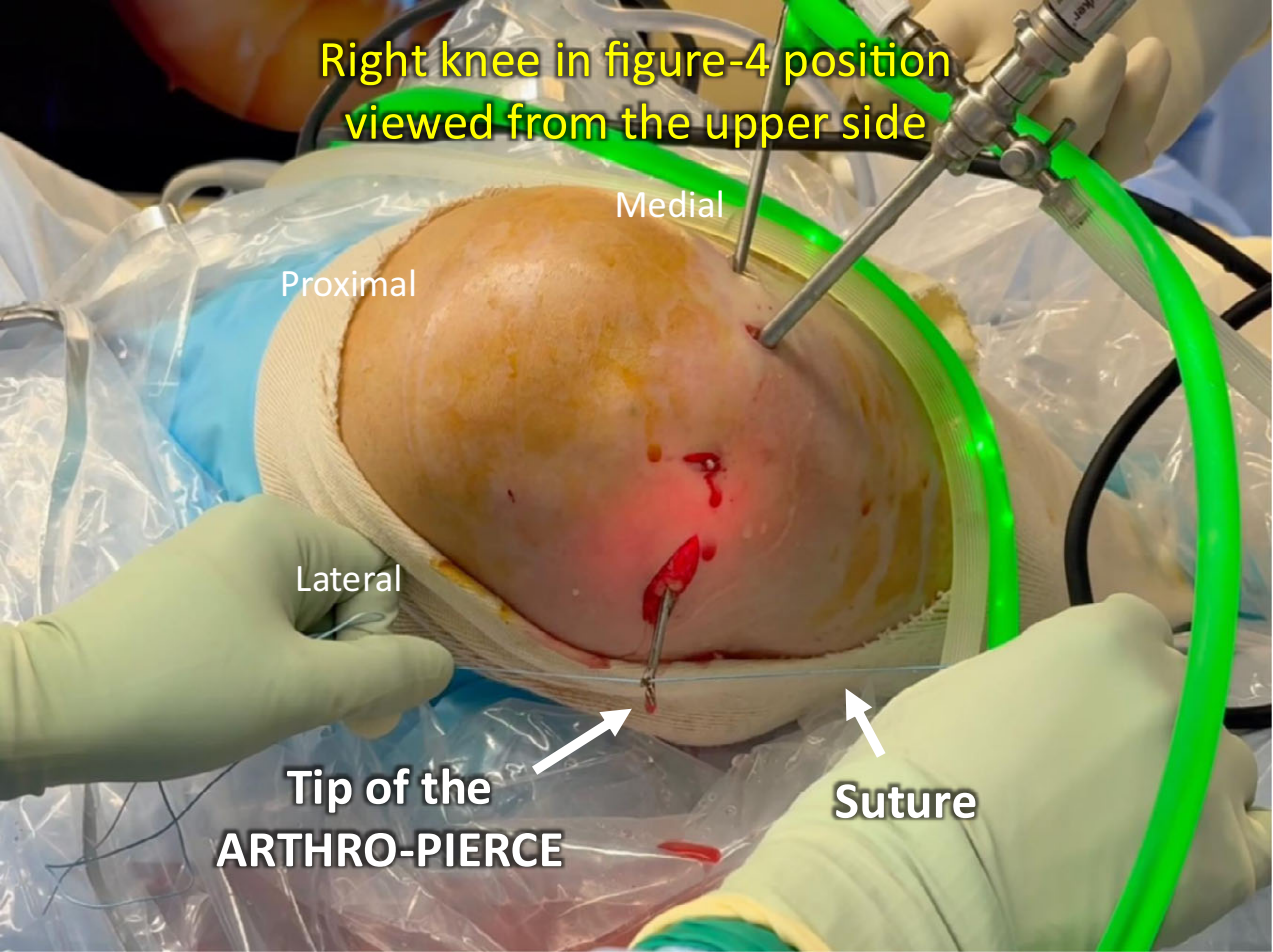
Suture gripping of the ARTHRO‐PIERCE (right knee, supine and figure‐4 position, view from the upper side). A skin incision is made near the suture exit point. The ULTRABRAID Suture is then grasped using the ARTHRO‐PIERCE in the extra‐articular space.

**FIGURE 6:**
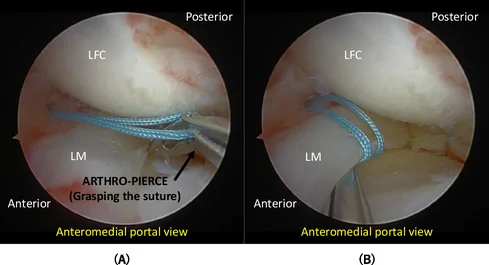
Second insertion of the ARTHRO‐PIERCE (right knee, supine and figure‐4 position, arthroscopic anteromedial portal views). (A) The ARTHRO‐PIERCE is withdrawn by gripping the ULTRABRAID Suture into the joint space. (B) The ARTHRO‐PIERCE is inserted, gripping the ULTRABRAID Suture into the meniscus and extra‐articular space. The ULTRABRAID Suture is then released into the extra‐articular space, and the ARTHRO‐PIERCE is withdrawn into the joint space. (LFC, lateral femoral condyle; LM, lateral meniscus.)

### Second Insertion of the ARTHRO‐PIERCE (While Gripping the Suture)

The ARTHRO‐PIERCE is inserted while gripping the suture into the meniscus and extra‐articular space (Figure 6B). The suture is then released, and the ARTHRO‐PIERCE is withdrawn into the joint space.

### Tensioning and Knot Tying

The suturing process is repeated until the desired sutures are completed (Figure 7A). The anterior portion of the LM is visualized from the anteromedial portal; the suture is then adjusted to the appropriate tension and tied (Figures 7B and 8).

**FIGURE 7:**
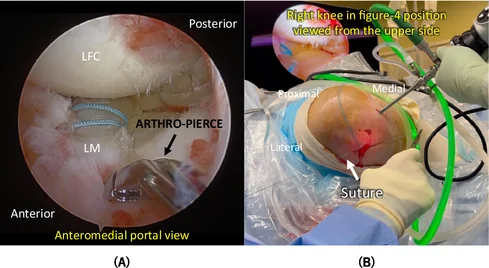
Completing suturing, tensioning, and knot tying (right knee, supine and figure‐4 position, arthroscopic anteromedial portal view and view from the upper side). (A) We performed 3 sutures in the anterior portion of the LM using the ULTRABRAID Suture. (B) The ULTRABRAID Suture is adjusted for appropriate tension and then tied. (LFC, lateral femoral condyle; LM, lateral meniscus.)

**FIGURE 8:**
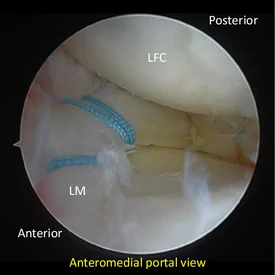
Postoperative arthroscopic view (right knee, supine and figure‐4 position, arthroscopic anteromedial portal view). Postoperatively, reduction of the LM and adequate stability can be confirmed. (LFC, lateral femoral condyle; LM, lateral meniscus.)

### Postoperative Management

The knee is immobilized in full extension in a brace for 1 week after the operation, during which nonweight‐bearing is implemented. From 1 week postoperatively, range‐of‐motion training with a limit of <90° is initiated, and from 4 weeks postoperatively, range‐of‐motion training without range restrictions is initiated. Full weight‐bearing is allowed 1 week postoperatively with a hinge brace.

## DISCUSSION

The SGT for meniscal repair was described to combine accurate intra‐articular needle placement with flexible extra‐articular suture handling, mitigating the key limitations of the conventional inside‐out, outside‐in, and all‐inside techniques.^4^, ^6^ In addition, this method imposes no restrictions on suture materials and is applicable to a wide range of suturing techniques, including various continuous suturing methods.^5^, ^7^, ^8^, ^9^, ^10^, ^11^ The present modification shows that ARTHRO‐PIERCE, although intended for hip arthroscopy, can function as an effective passer for SGT in the knee.

The principal technical advantages observed were related to device geometry and suture handling (Table 2). When a conventional suture shuttle holds a suture at its tip, the effective piercing profile may become relatively blunt. In this setting, the ARTHRO‐PIERCE, which directly grasps the suture with its jaws rather than relying on a loop mechanism, provides a functionally sharper tip profile during tissue penetration. This may facilitate penetration of the meniscocapsular tissue, particularly in the anterior segment where resistance is higher. A rigid shaft can improve trajectory control, which is particularly relevant for anterior horn suturing, where instrument deflection may limit reach. Additionally, the ability to directly grasp the suture may reduce the risk of suture loss or mechanical failure associated with a loop.

**TABLE 2:** Advantages and Limitations

Advantages of SGT With SS	Additional Advantages of ARTHRO‐PIERCE‐Assisted SGT	Limitations
Accurate needle insertion from inside the joint	Sharper functional tip profile during penetration compared with an SS holding a suture at its tip	May not be applicable to meniscal regions unreachable by the instrument even with accessory portals
Flexible withdrawal of the suture from outside the capsule	Rigid shaft improves reach and control for meniscal suturing	
No restrictions on the suturing technique (e.g., repair location and continuous suturing)	Robust suture grasping without reliance on a loop mechanism, reducing the risk of loop failure and suture loss	
No restrictions on suture caliber or material		
Extra‐articular knots minimize intra‐articular knot bulk and potential chondral abrasion		
Creation of far‐medial or far‐lateral portals enables suturing of the anterior portion of the meniscus		

SGT, suture‐gripping technique; SS, suture shuttle.

This technique retains the core benefits of SGT: it does not restrict suture material selection, allows accessory portal creation (e.g., far‐medial portal) to optimize the needle angle, and places knots extra‐articularly, thus avoiding intra‐articular knot impingement.

Nevertheless, limitations similar to those of other inside‐out approaches remain, including difficulty in reaching certain regions and the need for careful extra‐articular dissection to minimize soft tissue or neurovascular risk. Further clinical studies are warranted to evaluate the reproducibility and outcomes compared with those of standard SGT using a suture shuttle.

In conclusion, ARTHRO‐PIERCE‐assisted SGT can be performed reliably for meniscal repair, preserving the advantages of the SGT while enabling easier penetration, improved access to the anterior horn, and more secure suture control than a conventional suture shuttle.

## DISCLOSURES

The authors (R.S., N.M., T.N., T.H., K.K., M.N., S.K., H.M.) declare that they have no known competing financial interests or personal relationships that could have appeared to influence the work reported in this paper.
